# Combined Effects of Oligopeptides Isolated from *Panax ginseng* C.A. Meyer and *Ostrea gigas* Thunberg on Sexual Function in Male Mice

**DOI:** 10.3390/ijerph18052349

**Published:** 2021-02-28

**Authors:** Di Li, Jinwei Ren, Lixia He, Jingqin Sun, Peng Liu, Yong Li

**Affiliations:** 1 Department of Clinical Nutrition, Peking University People’s Hospital, Beijing 100044, China; lidipku@bjmu.edu.cn (D.L.); liupengpku@bjmu.edu.cn (P.L.); 2 Department of Nutrition and Food Hygiene, School of Public Health, Peking University, Beijing 100191, China; 0062046170@bjmu.edu.cn (J.R.); sunjingqin@ihcams.ac.cn (J.S.); 3 Division of Molecular and Cellular Oncology, Harvard Medical School, Boston, MA 02215, USA; lixia_he@dfci.harvard.edu

**Keywords:** ginseng, oyster, oligopeptide, sexual, male

## Abstract

Male sexual debility affects patients’ confidence and damages the relationship between the couples and thus affects the stability of the family. This study aimed to investigate the effects of oligopeptides isolated from ginseng and oyster (GOPs and OOPs), separately and in combination, on sexual function in male mice. In the first experiment, male mice were randomly divided into five groups: vehicle control group; whey protein (125.0 mg kg^−1^) group; and GOPs 62.5, 125.0, and 250.0 mg kg^−1^ groups. In the second experiment, male mice were randomly divided into five groups: vehicle control group, whey protein (160.0 mg kg^−1^) group, and OOPs 80.0, 160.0, and 320.0 mg kg^−1^ groups. In the third experiment, male mice were randomly divided into six groups: vehicle control group, whey protein (222.5 mg kg^−1^) group, and GOPs + OOPs 62.5 + 160.0, 62.5 + 320.0, and 125.0 + 160.0, 125.0 + 320.0 mg kg^−1^ groups. Test substances were given by gavage once a day for 30 days. The sexual behavior parameters, serum nitric oxide (NO), testosterone, cyclic guanosine monophosphate (cGMP), and phosphodiesterase-5 (PDE5) concentrations were detected. We found that GOPs at 250.0 mg kg^−1^ improved male sexual behavior, NO, and testosterone content, whereas GOPs at 62.5 and 125.0 mg kg^−1^ and OOPs at 80.0, 160, and 320 mg kg^−1^ did not have significant effects. The combination of 62.5 mg kg^−1^ GOPs + 160.0 mg kg^−1^ OOPs and the combination of 125.0 mg kg^−1^ GOPs + 320.0 mg kg^−1^ OOPs improved male sexual behavior, serum NO, testosterone, and cGMP contents and decreased PDE5 content. The combination of 62.5 mg kg^−1^ GOPs and 160.0 mg kg^−1^ OOPs had the best effects among four combined groups. These results suggested that GOPs in combination with OOPs had the synergistic effects of enhancing male sexual function, probably via elevating serum testosterone, NO, and corpus cavernosum cGMP level and decreasing the corpus cavernosum PDE5 level. GOPs and OOPs could be novel natural agents for improving male sexual function.

## 1. Introduction

Male sexual debility is caused by the degenerative diseases in testicles. Male sexual debility is diagnosed when the general level of blood testosterone is below 3 µg L^−1^ [1]. With the quickening pace of modern life, the fierce competition of society, drug abuse, speedy increase of the aged population, and a variety of other reasons, the morbidity of male sexual debility is increasing steadily. Although this disease does not endanger the patient’s life, it can seriously lower the quality of life of the patients, affect the patients’ confidence, and damage the relationship between couples and thus affect the stability of the family. Therefore, the research on male sexual debility is of great significance to promote personal health and social harmony and has become a hot research topic.

Currently, the treatment of male sexual debility is focused on drug therapy, including testosterone replacement therapy [2,3,4,5] and phosphodiesterase-5 inhibitor therapy [6,7,8,9,10]. However, these treatments have some limitations and have varying degrees of side effects [11,12,13]. In recent years, nutritional interventions have received increasing attention, and there have been numerous studies showing that natural food ingredients are safe and effective in improving sexual function.

Ginseng (*Panax ginseng* C.A. Meyer), a traditional Chinese medicinal herb, has been used as a restorative medicine for thousands of years [14]. In 2012, Ministry of Public Health of China approved ginseng as new resource food [15]. The ginseng root contains numerous bioactive components, such as ginsenoside, polysaccharides, amino acids, and peptides, that give it extensive biological activities including antioxidation [16,17,18], anti-hypoxia effect [19], immunoregulatory activity [20,21], hypolipidemic capacity [22,23], and the capacity to improve male sexual function [24,25,26,27]. Most studies attribute the effects of ginseng on male sexual function to ginsenoside [28,29]. *Ostrea gigas* Thunberg, also known as oyster, is rich in nutritional value and medicinal value. In 1758, the *Compendium of Materia Medica* recorded its medical applications in improving male sexual function [30]. Modern studies show that oysters can improve male sexual function by inhibiting the activity of phosphodiesterase-5 (PDE5) [31]. However, the absorption rate of whole ginseng roots and oysters in human intestines is very low after oral administration, while oligopeptides, with low molecular weight, absorbable features, and high bioavailability, exist in whole ginseng roots and oysters as an important bioactive component. However, the effects of oligopeptides isolated from ginseng and oyster (GOPs and OOPs) on sexual function in male mice are rarely reported. Thus, the present study was designed to investigate the effects of GOPs, OOPs, and their combination on sexual function in male mice. This study may provide an important theoretical basis for exploring novel natural agents that improve male sexual function and may contribute to the further development and utilization of traditional ingredients such as ginseng and oysters.

## 2. Materials and Methods

### 2.1. Preparation and Identification of GOPs and OOPs

The GOP and OOP samples were provided by SinoMed Peptide Valley Bioengineering Co., Ltd. and International Bioactive Peptides Institute. GOPs and OOPs were derived from the Jilin ginseng root and oyster meat by enzymatic hydrolysis, respectively (Patent: CN105154509A, CN107997184A). In brief, fresh ginseng roots and oyster meat were cleansed, minced, homogenized in distilled water, and treated by complex protease (3000 U/g protein) at 40 °C for 3 h after adjusting the pH to 8.0 by sodium hydroxide. Next, nanofiltration, cryoconcentration, decolorization, purification, and spray drying were performed to obtain GOP and OOP powders. The powders were stored in a refrigerator at 4 °C under vacuum before being further processed.

The oligopeptide samples were purified by high-performance liquid chromatography (Waters Corporation, Milford, MA, USA) using a Phenomenex C18 column (10 mm × 250 mm). The mobile phase was acetonitrile 0.05 mol/L phosphate buffer (pH 3.2) (10:90) with a flow rate of 2.0 mL/min monitored by a Water 486 tunable UV detector at 208 nm. The molecular weight distribution of the GOP and OOP samples was measured by LDI-1700 matrix-assisted laser desorption ionization–time-of-flight mass spectrometry (MALDI-TOF-MS, Liner Scientific Inc., Reno, NV, USA).

### 2.2. Chemicals and Reagents

The whey protein sample was provided by China National Research Institute of Food and Fermen, and whey protein content was 80%. Assay kits used for the determination of nitric oxide (NO), testosterone, cyclic guanosine monophosphate (cGMP), and PDE5 were purchased from Andy Huatai Technology Co. LTD (Beijing, China). All the reagents used in this study were of analytical grade.

### 2.3. Animals

A total of 320 adult male ICR mice (18–22 g) and 160 adult female ICR mice (14–19 g) were obtained from the Animal Service of Health Science Center, Peking University. The environment was maintained at 25 ± 1 °C with a relative humidity of 50–60% and a 12 h/12 h light–dark cycle. All the mice had free access to an AIN-93G diet and water. Mice were allowed 7 days to adapt to the new environment. All animals were treated according to the Principle of Laboratory Animal Care (National Institutes of Health Publication No. 85-23, revised 1985) and the guidelines of the Peking University Animal Research Committee.

### 2.4. Groups and Treatment

The first experiment was to explore the effect of GOPs on sexual function in male mice: Male mice were randomly divided into two subgroups (*n* = 50), namely subgroup A and subgroup B. Each subgroup was randomly divided into five groups (*n* = 10): vehicle control group (VCG), whey protein group 1 (125.0 mg kg^−1^, WPG1), and three groups of GOPs at different doses (62.5, 125.0, and 250.0 mg kg^−1^; GOPs-LG, GOPs-MG, and GOPs-HG, respectively). The VCG, WPG1, and three GOP groups were intragastrically administered vehicle, whey protein, and GOPs (0.1 mL/10 g), respectively. The animals were continuously treated for 30 days and then were used for further experiments.

The second experiment was to explore the effect of OOPs on sexual function in male mice: Male mice were randomly divided into two subgroups (*n* = 50), namely subgroup A and subgroup B. Each subgroup was randomly divided into five groups (*n* = 10): vehicle control group (VCG), whey protein group 2 (160.0 mg kg^−1^, WPG2), and three groups of OOPs at different doses (80.0, 160.0, and 320.0 mg kg^−1^; OOPs-LG, OOPs-MG, and OOPs-HG, respectively). The VCG, WPG2, and three OOP groups were intragastrically administered vehicle, whey protein, and OOPs (0.1 mL/10 g), respectively. The animals were continuously treated for 30 days and then were used for further experiments.

The third experiment was to explore the combined effects of GOPs and OOPs on sexual function in male mice: Male mice were randomly divided into two subgroups (*n* = 60), namely subgroup A and subgroup B. Each subgroup was randomly divided into six groups (*n* = 10): vehicle control group (VCG), whey protein group 3 (222.5 mg kg^−1^, WPG3), and four groups of GOPs + OOPs at different doses (62.5 + 160.0, 62.5 + 320.0, 125.0 + 160.0, and 125.0 + 320.0 mg kg^−1^; GOPs + OOPs 1, GOPs + OOPs 2, GOPs + OOPs 3, and GOPs + OOPs 4, respectively). The VCG, WPG2, and four GOP + OOP groups were intragastrically administered vehicle, whey protein, and GOPs + OOPs (0.1 mL/10 g), respectively. The animals were continuously treated for 30 days and then were used for further experiments.

### 2.5. Sexual Behavior Study

In the first experiment, sexual behavior of male mice in subgroup A was observed on 30th day of dosing, and intromission latency and intromission frequency were recorded. In the second and third experiments, sexual behavior of male mice in subgroup A was observed on 15th and 30th day of dosing, and mount latency, mount frequency, and intromission frequency were recorded. 

The detailed experimental method is as follows: Female mice were brought to estrus phase by administration of estradiol benzoate (250 μg/kg, i.h., administered 48 h prior to study; Sigma, St. Louis, MO, USA) and progesterone (250 μg/kg, i.h., administered 4 h prior to the study; Sigma) dissolved in corn oil. The sexual receptivity of female mice was evaluated before the study using a nonexperimental sexually vigorous male mouse [32]. One hour after dosing, male mice were introduced to the observation chamber (30 cm × 15 cm × 15 cm) and allowed to adapt to the environment for 5 min (one mouse per cage). Then by slowly lifting upper glass lid, a female mouse in estrus was randomly selected and introduced into the cage, and the behavior parameters were observed. Mount latency refers to the time taken by the male mouse up to the first mount on female mouse, intromission latency refers to the time taken by the male mouse up to the first intromission, mount frequency refers to the number of mounts by a male mouse on a female mouse in 20 min, intromission frequency refers to the number of intromissions by a male mouse in 20 min. The experiment was conducted under weak light and quiet condition between 20:00 and 23:00.

### 2.6. Determination of Serum NO and Testosterone

Thirty minutes after the final oral administration, the retro-orbital blood collection was used to collect blood sample from the mice in subgroup B. The serum was obtained by centrifugation at 3500 rpm at 4 °C for 10 min. The NO and testosterone content in serum were measured by detection kits according to the instructions by nitrate reductase and ELISA method, respectively.

### 2.7. Determination of Sex Organ Indexes

After the blood sample was obtained, the mice in subgroup B were sacrificed and the testis, epididymis, preputial glands, seminal vesicle and prostate glands were immediately isolated in an ice bath. Then the sex organs were weighed, and the organ indexes were calculated as weight of organ (g)/100 g body weight.

### 2.8. Examination of NO, cGMP, and PDE5 in Corpus Cavernosum Tissue

After the blood sample was obtained, the mice in subgroup B were sacrificed and the corpora cavernosa were immediately isolated in an ice bath. The concentrations of NO, cGMP and PDE5 were determined using available kits by Elisa method.

### 2.9. Statistical Analysis

The sexual behavior parameters are presented as mean ± standard error, and the other data are presented as mean ± standard deviation. Statistical significance with respect to vehicle was evaluated using one-way analysis of variance followed by Dunnett’s t-test, while the simple effects and interaction effects of GOPs and OOPs were evaluated by General Linear Model using SPSS software version 20 (SPSS Inc., Chicago, IL, USA). *p* < 0.05 was considered significant.

## 3. Results

### 3.1. Analysis of GOPs and OOPs

The contents of GOPs and OOPs were 95.42% and 80.23%, respectively; besides, both the relative molecular weights were less than 1000.

### 3.2. Effects of GOPs on Sexual Function in Male Mice

#### 3.2.1. Effects of GOPs on Sexual Behavior in Male Mice

**Figure 1:**
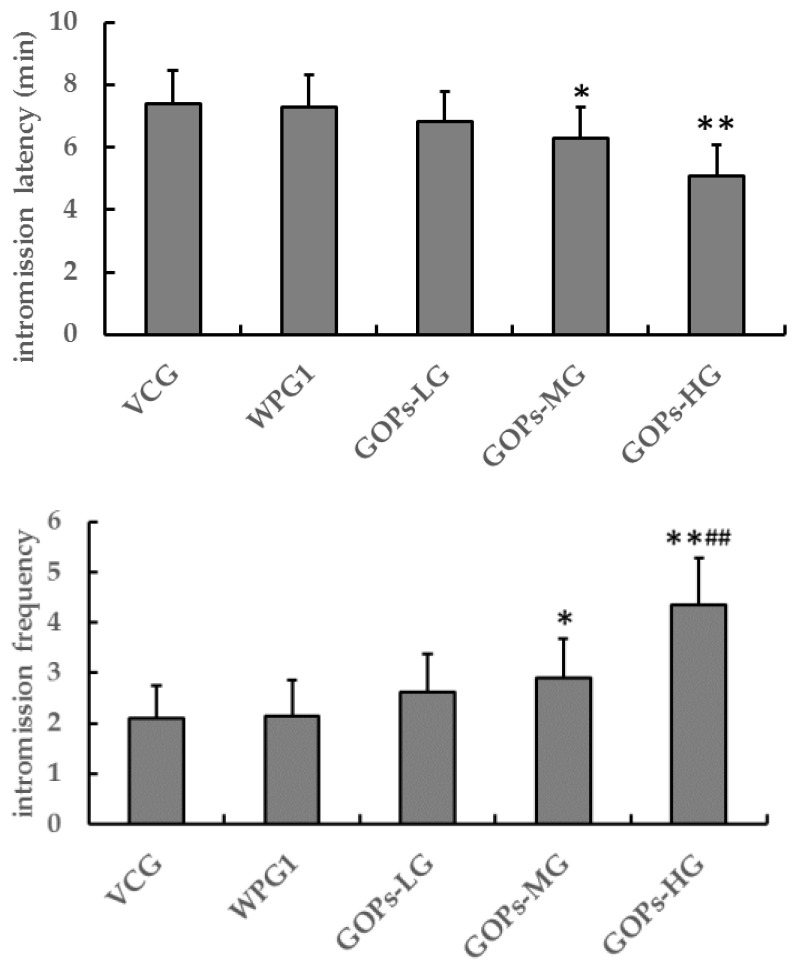
Effects of oligopeptides isolated from ginseng (GOPs) on sexual behavior in male mice. Data are presented as means ± SE (*n* = 10). * *p* < 0.05, ** *p* < 0.01 versus VCG; ^##^ *p* < 0.01 versus WPG1. VCG, vehicle control group; WPG1, whey protein 125.0 mg kg^−1^; GOPs-LG, 62.5 mg kg^−1^; GOPs-MG, 125.0 mg kg^−1^; GOPs-HG, 250.0 mg kg^−1^.

As shown in Figure 1, after 30 days of treatment, there were no significant differences in sexual behavior between VCG and WPG1 (*p* > 0.05). Compared with VCG, the mice in GOPs-MG and GOPs-HG showed shorter intromission latency (*p* < 0.05, *p* < 0.01) and higher intromission frequency (*p* < 0.05, *p* < 0.01). Compared with WPG1, the mice in GOPs-HG showed shorter intromission latency (*p* < 0.01) and higher intromission frequency (*p* < 0.01).

#### 3.2.2. Effects of GOPs on Sex Organ Indexes in Male Mice

**Figure 2:**
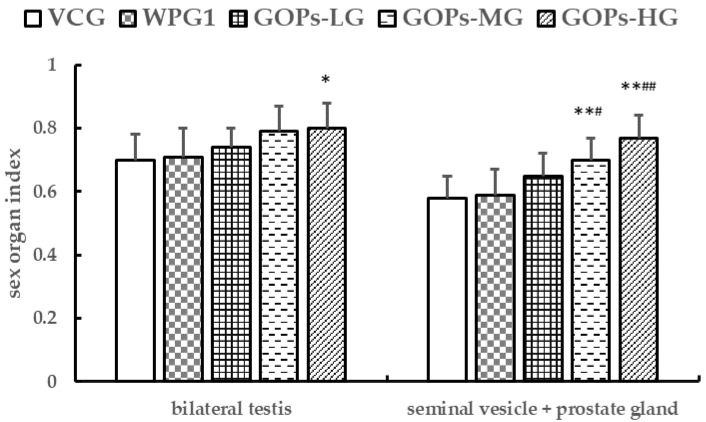
Effects of GOPs on sex organ indexes in male mice. Data are presented as means ± SD (*n* = 10). * *p* < 0.05, ** *p* < 0.01 versus VCG; ^#^ *p* < 0.05, ^##^ *p* < 0.01 versus WPG1. VCG, vehicle control group; WPG1, whey protein 125.0 mg kg^−1^; GOPs-LG, 62.5 mg kg^−1^; GOPs-MG, 125.0 mg kg^−1^; GOPs-HG, 250.0 mg kg^−1^.

After 30 days of treatment, there were no significant differences in sex organ indexes between VCG and WPG1 (*p* > 0.05). However, the bilateral testes index increased significantly in GOPs-HG in comparison with VCG (*p* < 0.05), and the seminal vesicle + prostate gland index increased significantly in GOPs-MG and GOPs-HG in comparison with VCG and WPG1 (*p* < 0.05, Figure 2).

#### 3.2.3. Effects of GOPs on Serum NO and Testosterone Contents in Male Mice

**Figure 3:**
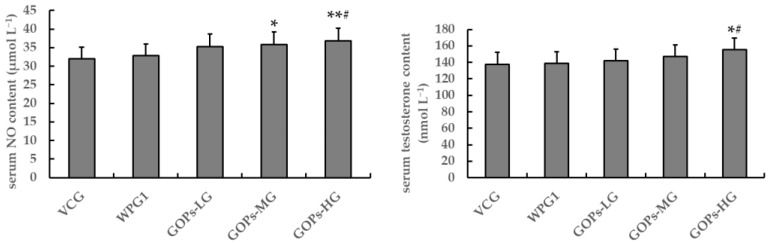
Effects of GOPs on serum NO and testosterone contents in male mice. Data are presented as means ± SD (*n* = 10). * *p* < 0.05, ** *p* < 0.01 versus VCG; ^#^ *p* < 0.05 versus WPG1. VCG, vehicle control group; WPG1, whey protein 125.0 mg kg^−1^; GOPs-LG, 62.5 mg kg^−1^; GOPs-MG, 125.0 mg kg^−1^; GOPs-HG, 250.0 mg kg^−1^.

After 30 days of treatment, no significant differences were observed in serum NO and testosterone contents between VCG and WPG1 (*p* > 0.05, Figure 3). Importantly, the mice in GOPs-MG had higher NO content than those in VCG (*p* < 0.05), and the mice in GOPs-HG had higher NO and testosterone contents than those in VCG and WPG1 (*p* < 0.05).

### 3.3. Effects of OOPs on Sexual Function in Male Mice

#### 3.3.1. Effects of OOPs on Sexual Behavior in Male Mice

**Figure 4:**
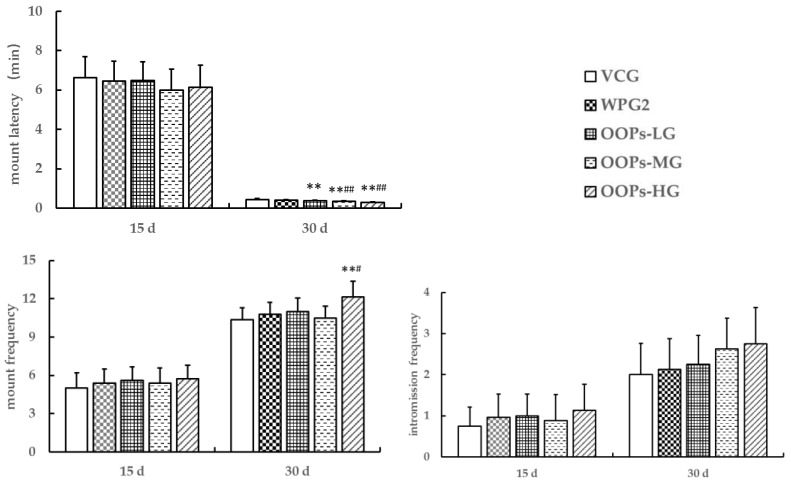
Effects of oligopeptides isolated from oyster (OOPs) on sexual behavior in male mice. Data are presented as means ± SE (*n* = 10). ** *p* < 0.01 versus VCG; ^#^ *p* < 0.05, ^##^ *p* < 0.01 versus WPG2. VCG, vehicle control group; WPG2, whey protein 160.0 mg kg^−1^; OOPs-LG, 80.0 mg kg^−1^; OOPs-MG, 160.0 mg kg^−1^; OOPs-HG, 320.0 mg kg^−1^.

We observed the sexual behavior in male mice treated with OOP for 15 and 30 days. After 15 days of treatment, the sexual behavior was not significantly different among all the groups (*p* > 0.05, Figure 4). After 30 days of treatment, the sexual behavior parameters were not significantly different between VCG and WPG2 (*p* > 0.05). Interestingly, the mount latency reduced as the dose of OOPs increased, and it decreased significantly in three OOP groups in comparison with VCG (*p* < 0.01) and showed significant differences in OOPs-MG and OOPs-HG in comparison with WPG2 (*p* < 0.01). The mice in OOPs-HG had higher mount frequency than those in VCG and WPG2 (*p* < 0.01, *p* < 0.05). The intromission frequency increased as the dose of OOPs rose but showed no significant differences among the groups (*p* > 0.05).

#### 3.3.2. Effects of OOPs on Sex Organ Indexes in Male Mice

**Figure 5:**
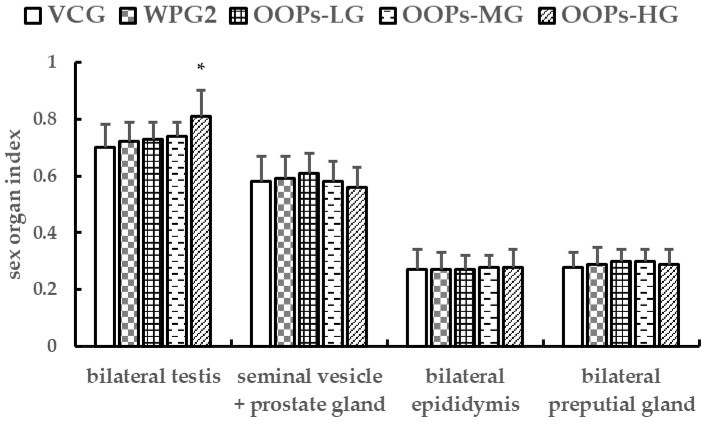
Effects of OOPs on sex organ indexes in male mice. Data are presented as means ± SD (*n* = 10). * *p* < 0.05 versus VCG. VCG, vehicle control group; WPG2, whey protein 160.0 mg kg^−1^; OOPs-LG, 80.0 mg kg^−1^; OOPs-MG, 160.0 mg kg^−1^; OOPs-HG, 320.0 mg kg^−1^.

Figure 5 shows that there were no significant differences in sex organ indexes between VCG and WPG2 after 30 days of treatment either (*p* > 0.05). Nevertheless, the bilateral testes index rose as the dose of OOPs increased, and it increased significantly in GOPs-HG in comparison with VCG (*p* < 0.05) while showing no significant differences in comparison with WPG2 (*p* > 0.05). The bilateral epididymis index, bilateral preputial gland index, and seminal vesicle + prostate gland index were not significantly different among all the groups (*p* > 0.05).

#### 3.3.3. Effects of OOPs on NO and Testosterone Contents in Male Mice

**Figure 6:**
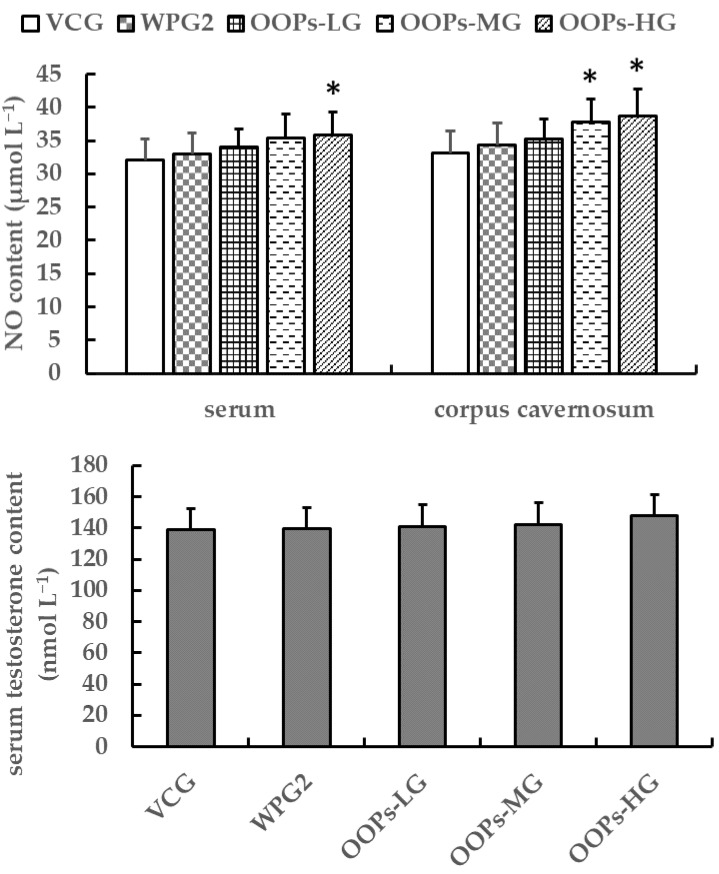
Effects of OOPs on NO and testosterone contents in male mice. Data are presented as means ± SD (*n* = 10). * *p* < 0.05 versus VCG. VCG, vehicle control group; WPG2, whey protein 160.0 mg kg^−1^; OOPs-LG, 80.0 mg kg^−1^; OOPs-MG, 160.0 mg kg^−1^; OOPs-HG, 320.0 mg kg^−1^.

From Figure 6, we can see that the serum testosterone content was not significantly different among all the groups after 30 days of treatment (*p* > 0.05). The NO content both in serum and corpus cavernosum showed no significant differences between VCG and WPG2 (*p* > 0.05) but increased as the dose of OOPs rose. In particular, the serum NO content in OOPs-MG and the corpus cavernosum NO content in OOPs-MG and OOPs-HG were higher than that in VCG (*p* < 0.05) while not significantly different in comparison with WPG2 (*p* > 0.05).

### 3.4. Combined Effects of GOPs and OOPs on Sexual Function in Male Mice

#### 3.4.1. Combined Effects of GOPs and OOPs on Sexual Behavior in Male Mice

**Figure 7:**
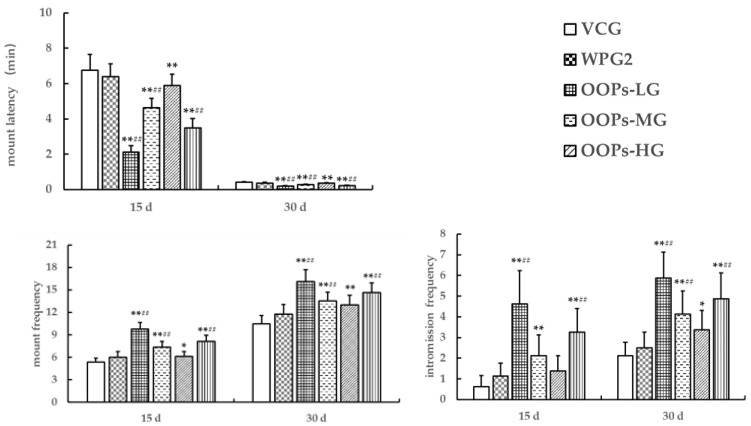
Combined effects of GOPs and OOPs on sexual behavior in male mice. Data are presented as means ± SE (*n* = 10). * *p* < 0.05, ** *p* < 0.01 versus VCG; ^##^ *p* < 0.01 versus WPG3. VCG, vehicle control group; WPG3, whey protein 222.5 mg kg^−1^; GOPs + OOPs 1, 62.5 + 160.0 mg kg^−1^; GOPs + OOPs 2, 62.5 + 320.0 mg kg^−1^; GOPs + OOPs 3, 125.0 + 160.0 mg kg^−1^; GOPs + OOPs 4, 125.0 + 320.0 mg kg^−1^.

After 15 and 30 days of treatment, the sexual behavior parameters in WPG3 were not significantly changed compared to VCG (*p* > 0.05). The shorter mount latency and increased mount frequency were found in the mice in all the GOP + OOP groups after 15 and 30 days of treatment in comparison with WPG3 (*p* < 0.05). In addition, the mice in all the GOP + OOP groups except GOPs + OOPs 3 had shorter mount latency and higher mount frequency than those in WPG3 after 15 and 30 days of treatment (*p* < 0.01). The intromission frequency in GOPs + OOPs 1, GOPs + OOPs 2, and GOPs + OOPs 4 was higher than that in VCG (*p* < 0.01), and increased intromission frequency was found in GOPs + OOPs 1 and GOPs + OOPs 4 in comparison with WPG3 (*p* < 0.01) after 15 and 30 days of treatment. Meanwhile, the intromission frequency in GOPs + OOPs 2 was higher than that in WPG3 (*p* < 0.01), and it was also increased in GOPs + OOPs 3 in comparison with VCG (*p* < 0.05) after 30 days of treatment (Figure 7).

Furthermore, we also found that GOPs and OOPs had significant interaction effects on all the sexual behavior parameters on day 15 and day 30 (*p* = 0.000). In Table 1, the results of simple effects analysis showed that GOPs had significant simple effects on all the sexual behavior parameters on day 15 and day 30 when the dose of OOPs was 160 mg kg^−1^ (*p* = 0.000). From Table 2 we can see when the dose of OOPs was 320 mg kg^−1^, GOPs had significant simple effects on the mount latency on day 15 and day 30 (*p* = 0.000, *p* = 0.025) but had no significant simple effects on the mount frequency and intromission frequency on day 15 and day 30 (*p* > 0.05). In addition, OOPs had significant simple effects on all the sexual behavior parameters when the dose of GOPs was 62.5 mg kg^−1^ (*p* < 0.01) or 125.0 mg kg^−1^ (*p* < 0.05).

#### 3.4.2. Combined Effects of GOPs and OOPs on Sex Organ Indexes in Male Mice

**Figure 8:**
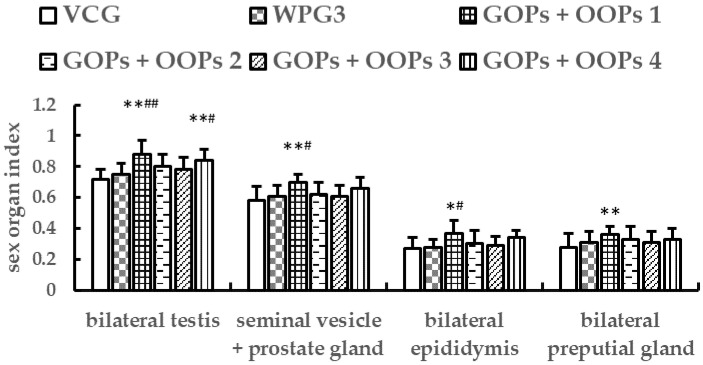
Combined effects of GOPs and OOPs on sex organ indexes in male mice. Data are presented as means ± SD (*n* = 10). * *p* < 0.05, ** *p* < 0.01 versus VCG; ^#^ *p* < 0.05, ^##^ *p* < 0.01 versus WPG3. VCG, vehicle control group; WPG3, whey protein 222.5 mg kg^−1^; GOPs + OOPs 1, 62.5 + 160.0 mg kg^−1^; GOPs + OOPs 2, 62.5 + 320.0 mg kg^−1^; GOPs + OOPs 3, 125.0 + 160.0 mg kg^−1^; GOPs + OOPs 4, 125.0 + 320.0 mg kg^−1^.

As shown in Figure 8, there were no significant changes in sex organ indexes of mice in WPG3 compared with VCG after 30 days of treatment (*p* > 0.05). All the sex organ indexes in GOPs + OOPs 1 increased significantly in comparison with VCG (*p* < 0.01, *p* < 0.01, *p* < 0.05, *p* < 0.01), meanwhile, all the sex organ indexes except bilateral preputial gland index increased significantly in comparison with WPG3 (*p* < 0.01, *p* < 0.05, *p* < 0.05). The bilateral testes index in GOPs + OOPs 4 was higher than that in VCG and WPG3 (*p* < 0.01, *p* < 0.05).

In addition, we found that GOPs and OOPs had no interaction effects on bilateral epididymis index and bilateral preputial gland index (*p* = 0.071, *p* = 0.181) but had significant interaction effects on bilateral testes index and seminal vesicle + prostate gland index (*p* = 0.044, *p* = 0.033). Therefore, we performed the simple effect analysis and found that GOPs had significant simple effects on bilateral testes index and seminal vesicle + prostate gland index when the dose of OOPs was 160 mg kg^−1^ (*p* = 0.045, *p* = 0.027) but had no significant simple effects when the dose of OOPs was 320 mg kg^−1^ (*p* = 0.382, *p* = 0.404) (Table 3). Furthermore, OOPs had significant simple effects on seminal vesicle + prostate gland index (*p* = 0.048) but had no significant simple effects on bilateral testes index (*p* = 0.098) when the dose of GOPs was 62.5 mg kg^−1^. When the dose of GOPs was 125.0 mg kg^−1^, OOPs had no significant simple effects both on bilateral testes index and seminal vesicle + prostate gland index (*p* = 0.209, *p* = 0.270) (Table 4).

#### 3.4.3. Combined Effects of GOPs and OOPs on NO and Testosterone Contents in Male Mice

**Figure 9:**
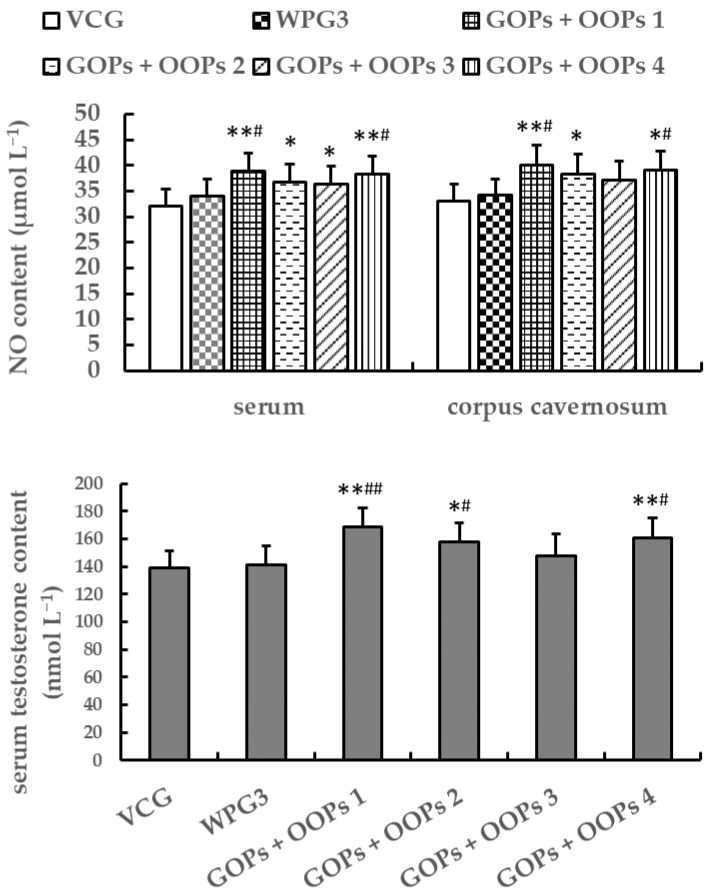
Combined effects of GOPs and OOPs on NO and testosterone content in male mice. Data are presented as means ± SD (*n* = 10). * *p* < 0.05, ** *p* < 0.01 versus VCG; ^#^ *p* < 0.05, ^##^ *p* < 0.01 versus WPG3. VCG, vehicle control group; WPG3, whey protein 222.5 mg kg^−1^; GOPs + OOPs 1, 62.5 + 160.0 mg kg^−1^; GOPs + OOPs 2, 62.5 + 320.0 mg kg^−1^; GOPs + OOPs 3, 125.0 + 160.0 mg kg^−1^; GOPs + OOPs 4, 125.0 + 320.0 mg kg^−1^.

After 30 days of treatment, the testosterone and NO contents showed no significant differences between VCG and WPG3 (*p* > 0.05, Figure 9). The serum testosterone content of mice in GOPs + OOPs 1, GOPs + OOPs 2, and GOPs + OOPs 4 was higher than that in VCG and WPG3 (*p* < 0.05). The serum and corpus cavernosum NO contents of mice in GOPs + OOPs 1 and GOPs + OOPs 4 were higher than those in VCG and WPG3 (*p* < 0.05). The NO content in GOPs + OOPs 2 and GOPs + OOPs 3 increased in serum in comparison with VCG (*p* < 0.05), and increased corpus cavernosum NO content was found in GOPs + OOPs 3 when compared to VCG (*p* < 0.05).

We also found that GOPs and OOPs had no interaction effects on serum and corpus cavernosum NO contents (*p* = 0.134, *p* = 0.298) but had significant interaction effects on serum testosterone content (*p* = 0.049). As shown in Table 5, GOPs had significant simple effects on serum testosterone content when the dose of OOPs was 160 mg kg^−1^ (*p* = 0.016) but had no significant simple effects when the dose of OOPs was 320 mg kg^−1^ (*p* = 0.739). In addition, OOPs had no significant simple effects on serum testosterone content when the dose of GOPs was 62.5 mg kg^−1^ (*p* = 0.189) or 125.0 mg kg^−1^ (*p* = 0.127) (Table 6).

#### 3.4.4. Combined Effects of GOPs and OOPs on Corpus Cavernosum cGMP and PDE5 Content in Male Mice

**Figure 10:**
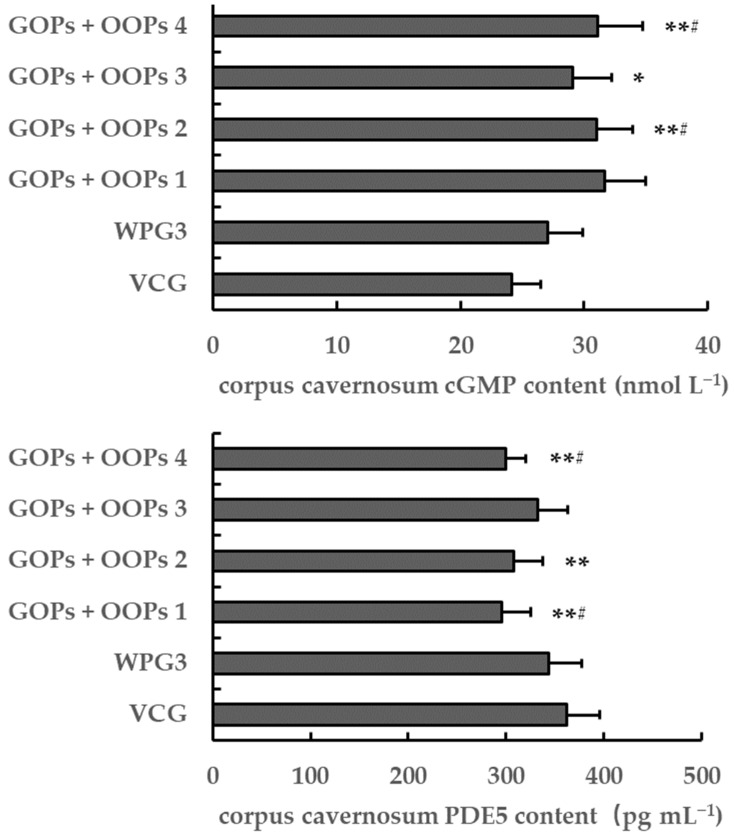
Combined effects of GOPs and OOPs on corpus cavernosum cGMP and PDE5 content in male mice. Data are presented as means ± SD (*n* = 10). * *p* < 0.05, ** *p* < 0.01 versus VCG; ^#^ *p* < 0.05 versus WPG3. VCG, vehicle control group; WPG3, whey protein 222.5 mg kg^−1^; GOPs + OOPs 1, 62.5 + 160.0 mg kg^−1^; GOPs + OOPs 2, 62.5 + 320.0 mg kg^−1^; GOPs + OOPs 3, 125.0 + 160.0 mg kg^−1^; GOPs + OOPs 4, 125.0 + 320.0 mg kg^−1^.

No significant differences were found in the corpus cavernosum cGMP and PDE5 content between VCG and WPG3 after 30 days of treatment (*p* > 0.05, Figure 10). However, the corpus cavernosum cGMP content in GOPs + OOPs 1, GOPs + OOPs 2, and GOPs + OOPs 4 was higher than that in VCG and WPG3 (*p* < 0.01, *p* < 0.05), and increased corpus cavernosum cGMP content was found in GOPs + OOPs 3 in comparison with VCG (*p* < 0.05). Additionally, the corpus cavernosum PDE5 content in GOPs + OOPs 1 and GOPs + OOPs 4 was significantly lower than that in VCG and WPG3 (*p* < 0.01, *p* < 0.05), and it was decreased significantly in GOPs + OOPs 2 compared with VCG (*p* < 0.05).

Nevertheless, GOPs and OOPs had no interaction effects on corpus cavernosum cGMP and PDE5 content (*p* = 0.366, *p* = 0.094).

## 4. Discussion

With the fast development of society and economy, humans’ demands for better quality of life are becoming increasingly urgent. However, mental and physical exhaustion, tobacco use, and excessive alcohol consumption caused by the quickening pace of modern lives and fierce competition of society are driving an increase in the morbidity of male sexual debility [33]. In the present study, we evaluated the effects of GOPs and OOPs on sexual function in male mice for the first time. Moreover, the combined effects of GOPs and OOPs were also further explored.

Whey protein is a catch-all term that includes several protein fractions such as β-lactoglobulin, α-lactalbumin, and immunoglobulin. Because it is easy to digest and absorb, whey protein has high bioavailability and various biological activities including immune support; fatigue resistance; and antioxidant, antibacterial, and antiviral activities [15]. To exclude false-positive results caused by protein intake, we used whey protein as protein control by comparing oligopeptides to whey protein. According to the results we obtained from the current study, the effects of whey protein on sex function were not observed under our conditions, suggesting that the effects were due to oligopeptides, not protein intake.

Male sexual function includes sexual desire, penile erection, sexual intercourse, orgasm, and ejaculation [34]. Mounting is a measure of sexual motivation, whereas intromission is a reflection of the facilitation of sexual motivation [35]. In the present study, GOPs at 250.0 mg kg^−1^ showed the effects of decreasing intromission latency and increasing in intromission frequency, whereas GOPs at 62.5 or 125.0 mg kg^−1^ had no significant effects on intromission latency and intromission frequency. Besides, we observed a decrease in mount latency in mice treated with OOPs at 160 or 320 mg kg^−1^ and an increase in mount frequency in mice treated with OOPs at 320 mg kg^−1^ but no increase in intromission frequency in mice treated with OOPs at 80.0, 160, or 320 mg kg^−1^. These findings suggest that GOPs at 250.0 mg kg^−1^ but not GOPs at 62.5 or 125.0 mg kg^−1^ might improve male sexual function, and OOPs at 160 or 320 mg kg^−1^ might only improve sexual motivation. In addition, mice treated with GOPs + OOPs at 62.5 + 160.0, 62.5 + 320.0, or 125.0 + 320.0 mg kg^−1^ showed shorter mount latency and higher mount frequency, meanwhile, mice treated with GOPs + OOPs at 62.5 + 160.0 or 125.0 + 320.0 mg kg^−1^ showed higher intromission frequency compared with the whey protein group on day 15 and day 30. It thus appeared that GOPs + OOPs at 62.5 + 160.0 or 125.0 + 320.0 mg kg^−1^ might improve male sexual function, and GOPs + OOPs at 62.5 + 320.0 mg kg^−1^ might only improve sexual motivation.

Studies on reproductive physiology and reproductive endocrinology have shown that the sexual activity is regulated by the neuroendocrine system, especially sexual hormone levels [36]. Testosterone is an important sex hormone in the growth and development of the body, affecting the growth and development of germ cells as well as the development of sexual organs [37]. The testicle is the most important organ for generating testosterone in mice, and testicular interstitial cells are the main synthetic cells. In the present study, GOPs at 250.0 mg kg^−1^ resulted in an increase in the concentrations of serum testosterone, whereas GOPs at 62.5 or 125.0 mg kg^−1^ and OOPs at 80.0, 160, or 320 mg kg^−1^ had no significant increase. Besides, we observed an increase in bilateral testes index in mice treated with GOPs at 250.0 mg kg^−1^ and OOPs at 320 mg kg^−1^. These findings suggest that GOPs at 250.0 mg kg^−1^ could elevate testosterone level, and OOPs at 320 mg kg^−1^ might have the potential to elevate testosterone level. Furthermore, mice treated with GOPs + OOPs at 62.5 + 160.0, 62.5 + 320.0, or 125.0 + 320.0 mg kg^−1^ showed an increase in the concentration of serum testosterone; meanwhile, mice treated with GOPs + OOPs at 62.5 + 160.0 or 125.0 + 320.0 mg kg^−1^ showed an increase in bilateral testes index.

Penile erection is an important component of male sexual function, the mechanism of which is related to many neurotransmitters, ions, and enzymes [38]. After diffusing into corpus cavernosum smooth muscle (CCSM), NO can stimulate soluble guanylyl cyclase (sGC) to generate cGMP from guanosine triphosphate (GTP). cGMP relaxes the CCSM by opening the potassium ion channel, thus increasing penis blood flow and erecting the penis [7,39]. Therefore, NO and cGMP are beneficial for penile erection. In the present study, mice treated with GOPs at 250.0 mg kg^−1^ showed an increase in the concentrations of serum NO, whereas those treated with GOPs at 62.5 or 125.0 mg kg^−1^ and OOPs at 80.0, 160, or 320 mg kg^−1^ had no significant increase. Besides, mice treated with GOPs + OOPs at 62.5 + 160.0 or 125.0 + 320.0 mg kg^−1^ showed an increase in the concentrations of NO both in serum and corpus cavernosum. In short, GOPs at 62.5 or 125.0 mg kg^−1^ and OOPs at 80.0, 160, or 320 mg kg^−1^ had no effects on NO content, but GOPs + OOPs at 62.5 + 160.0 or 125.0 + 320.0 mg kg^−1^ did have effects on NO content. Moreover, we observed an increase in corpus cavernosum cGMP content in mice treated with GOPs + OOPs at 62.5 + 160.0, 62.5 + 320.0, or 125.0 + 320.0 mg kg^−1^, which might explain the enhancement to penile erection.

Phosphodiesterases (PDEs) are a multigene family, among which PDE5 is closely related to the reproductive system and is mainly distributed in the corpus cavernosum and platelets. PDE5 specially hydrolyses cGMP [7,40,41]. Therefore, PDE5 reduces the function of cGMP to relax CCSM, thus affecting penile erection. In the present study, mice treated with GOPs + OOPs at 62.5 + 160.0 or 125.0 + 320.0 mg kg^−1^ showed a decrease in the corpus cavernosum PDE5 content, suggesting that these two dosages might be beneficial for penile erection.

Moreover, we analyzed the interaction effects of GOPs and OOPs. The results showed that GOPs and OOPs had interaction effects on bilateral testes index, seminal vesicle + prostate gland index, mount latency, mount frequency, intromission frequency, and serum testosterone content. The subsequent simple effects analysis results showed that there was significant difference between GOPs at 62.5 mg kg^−1^ and 125.0 mg kg^−1^ on the above variables when the dosage of OOPs was 160 mg kg^−1^. Interestingly, among the four combined groups, the best treatment for enhancing male sexual function was GOPs + OOPs at 62.5 + 160.0 mg kg^−1^, which obviously was the lowest dosage match.

## 5. Conclusions

In conclusion, the present study indicated that GOPs at 250.0 mg kg^−1^ had the effects of enhancing male sexual function, whereas GOPs at 62.5 or 125.0 mg kg^−1^ and OOPs at 80.0, 160, or 320 mg kg^−1^ did not have such effects but might have the potential to elevate male sexual function. Besides, GOPs + OOPs at 62.5 + 160.0 or 125.0 + 320.0 mg kg^−1^ had the effects of enhancing male sexual function, while GOPs + OOPs at 62.5 + 320.0 mg kg^−1^ might have such effects. Moreover, GOPs + OOPs at 62.5 + 160.0 mg kg^−1^ was the best treatment combination for enhancing male sexual function and thus is of great economic value and application prospect. This study provides an important theoretical basis for exploring novel natural agents that improve male sexual function and contributes to the further development and utilization of traditional ingredients. Further in vitro studies should be conducted to explain the exact mechanism in enhancing male sexual function.

## Figures and Tables

**Table 1:** Simple effects of GOPs on sexual behavior parameters (*p* value).

OOPs (mg kg^−1^)	Mount Latency		Mount Frequency		Intromission Frequency	
	15 days	30 days	15 days	30 days	15 days	30 days
160.0	0.000	0.000	0.000	0.000	0.000	0.000
320.0	0.000	0.025	0.065	0.106	0.064	0.200

**Table 2:** Simple effects of OOPs on sexual behavior parameters (*p* value).

GOPs (mg kg^−1^)	Mount Latency		Mount Frequency		Intromission Frequency	
	15 days	30 days	15 days	30 days	15 days	30 days
62.5	0.000	0.002	0.000	0.001	0.000	0.005
125.0	0.000	0.000	0.000	0.023	0.003	0.014

**Table 3:** Simple effects of GOPs on sex organ indexes (*p* value).

OOPs (mg kg^−1^)	Bilateral Testes Index	Seminal Vesicle + Prostate Gland Index
160.0	0.045	0.027
320.0	0.382	0.404

**Table 4:** Simple effects of OOPs on sex organ indexes (*p* value).

GOPs (mg kg^−1^)	Bilateral Testes Index	Seminal Vesicle + Prostate Gland Index
62.5	0.098	0.048
125.0	0.209	0.270

**Table 5:** Simple effects of GOPs on serum testosterone content.

OOPs (mg kg^−1^)	*p*
160.0	0.016
320.0	0.739

**Table 6:** Simple effects of OOPs on serum testosterone content.

GOPs (mg kg^−1^)	*p*
62.5	0.189
125.0	0.127

## Data Availability

Raw data are stored on a computer in the office of the corresponding author for five years and can be made available upon request.
